# Abundant neutrophil extracellular traps in thrombus of patient with microscopic polyangiitis

**DOI:** 10.3389/fimmu.2012.00333

**Published:** 2012-11-12

**Authors:** Daigo Nakazawa, Utano Tomaru, Chiho Yamamoto, Satoshi Jodo, Akihiro Ishizu

**Affiliations:** ^1^Department of Internal Medicine II, Hokkaido University HospitalSapporo, Japan; ^2^Department of Pathology, Hokkaido University Graduate School of MedicineSapporo, Japan; ^3^Department of Internal Medicine, Tomakomai City HospitalTomakomai, Japan; ^4^Faculty of Health Sciences, Hokkaido UniversitySapporo, Japan

**Keywords:** MPO-ANCA, microscopic polyangiitis, neutrophil extracellular traps, deep vein thrombosis, histone-citrullination

## Abstract

This is a case study of a patient diagnosed with microscopic polyangiitis (MPA) and complicated with deep vein thrombosis (DVT), who died of respiratory failure despite treatment. Autopsy revealed severe crescentic glomerulonephritis and massive alveolar hemorrhage. The thrombus contained abundant neutrophils. Although it is reported that patients with ANCA-associated vasculitis (AAV) have an increased risk of DVT, it remains elusive why they are prone to thrombosis. A recent study has demonstrated the presence of neutrophil extracellular traps (NETs), a newly recognized mode of neutrophil cell-death, in glomerular crescents of MPA patients. Interestingly, NETs were identified in the thrombus as well as in the glomerular crescents in the present case. When compared to other thrombi unrelated to MPA, the amount of NETs was significantly greater in the MPA patient. On the other hand, NETs are critically involved in thrombogenesis because histones within NETs can bind platelets and blood coagulants. Although this is important in regard to containment of microbes within NETs, excessive NETs could cause thrombosis. The collective findings suggest the possibility that thrombosis could be critically associated with MPA via NETs, and that NETs could be a therapeutic target in MPA patients.

## Case presentation

A 56-years-old woman was admitted to the section of Internal Medicine because of fever and tender swelling of the left leg that began 2 weeks ago. Urinalysis revealed microhematuria (30–49/high power field) and proteinuria (100–300 mg/dl). Hematological examinations showed leukocytosis with white blood cell counts of 16,410/μl, anemia with hemoglobin of 6.9 g/dl, and normal platelet counts of 24.0 × 10^4^/μl. Blood chemistry demonstrated elevated levels of blood urea nitrogen (22.9 mg/dl) and creatinine (2.85 mg/dl). The serum level of C-reactive protein was also elevated (7.39 mg/dl). Myeloperoxidase-anti-neutrophil cytoplasmic antibody (MPO-ANCA) was positive (836 units/ml); while, other autoantibodies, including proteinase 3-ANCA, anti-glomerular basement membrane antibody, and anti-phospholipid antibody, were negative. In coagulation tests, fibrin degradation product*s* and D-dimers were markedly elevated (28.1 μg/ml and 21.7 μg/ml, respectively). Contrast-enhanced computed tomography showed bilateral infiltrative shadows in the lower lobules of the lungs with pleural effusion and thrombosis in the left common iliac vein. Based on these findings, the patient was diagnosed with microscopic polyangiitis (MPA) complicated with pneumonia and deep vein thrombosis (DVT). Immediately, a filter was inserted into the inferior vena cava in order to prevent fatal pulmonary embolism. Although alveolar hemorrhage was considered to be a differential diagnosis for pneumonia, antibiotic treatment was initiated because bacterial infection could not be ruled out at this time. However, she developed dyspnea and hemoptysis 5 days later. Bronchoscopy and bronchoalveolar lavage were not conducted because of the respiratory distress; however, alveolar hemorrhage due to MPA was still considered. Therefore, combination therapy of corticosteroid (intravenous administration of 0.5 g methylprednisolone for 3 consecutive days followed by oral administration of 40 mg/day prednisolone) and cyclophosphamide (intravenous administration of 500 mg/day cyclophosphamide) was started 5 days after the admission. However, she died of respiratory failure 4 days later.

**Figure 1:**
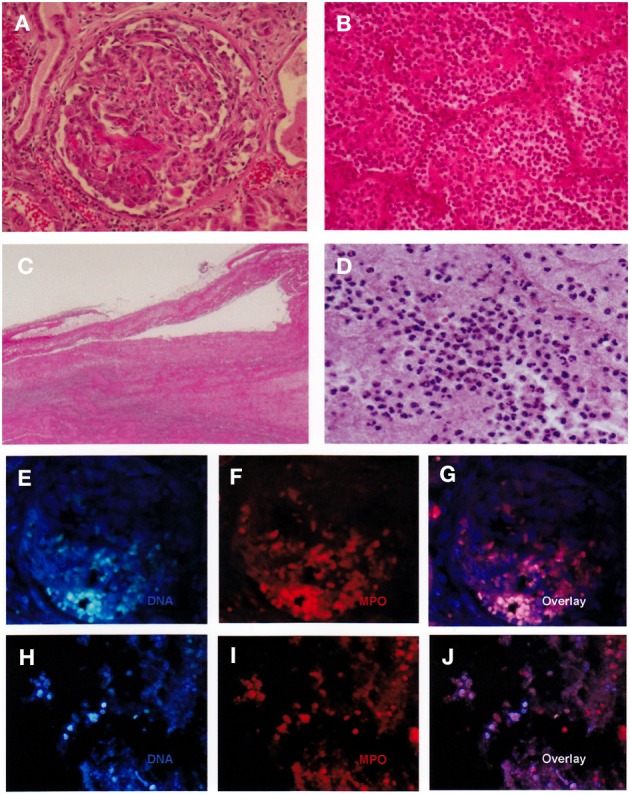
**Autopsy findings. (A)** Crescentic necrotizing glomerulonephritis. **(B)** Alveolar hemorrhage with neutrophil infiltration. **(C, D)** DVT: neutrophils were abundant in the thrombus. **(E–G)** NETs in the glomerulus. Blue: DNA stained by DAPI. Red: MPO. NETs were present in the crescent. **(H–J)** NETs in the thrombus. The detection of NETs was performed similar to the renal specimens. Original magnification: ×40 **(C)**, ×200 **(B)**, × 400 **(A, D, E–J)**.

Autopsy revealed diffuse crescentic necrotizing glomerulonephritis without immunoglobulin deposition and massive alveolar hemorrhage with neutrophil infiltration (Figures 1A,B). The glomerular findings were consistent with pauci-immune crescentic glomerulonephritis of MPA. Alveolar hemorrhage was also considered as a sign of MPA, though typical capillaritis could not be identified in the lungs. The thrombus was relatively, fresh and contained abundant neutrophils (Figures 1C,D). In the thrombus, no microbe was detected by special staining techniques, including Gram stain, Giemsa stain, and Periodic acid-Schiff reaction.

## Background

MPA is an ANCA-associated vasculitis (AAV), in which pauci-immune crescentic glomerulonephritis develops with generation of MPO-ANCA. Alveolar hemorrhage due to capillaritis in the lungs is a frequent complication and is sometimes fatal. It is reported that AAV patients have an increased risk of developing DVT, especially during the active stage of the disease (Stassen et al., 2008). Vasculitis possibly triggers thrombosis through the action of inflammatory cytokines and other substances related to the injury of vascular endothelial cells. However, the formation of thrombus does not always occur in the affected vessels. Thus, it remains elusive why AAV patients are prone to thrombosis.

A recent study has demonstrated the presence of neutrophil extracellular traps (NETs), a newly recognized mode of neutrophil cell-death, in glomerular crescents of MPA patients (Kessenbrock et al., 2009). Kessenbrock et al. suggested that MPO-ANCA could bind with activated neutrophils and accelerate NETs formation. Intrinsically, NETs play roles in the innate immune response to microbes, in which the meshwork is composed of DNA fibers that comprise histones and antimicrobial proteins including MPO (Brinkmann et al., 2004). Under physiological condition, NETs are induced following phagocytosis in order to trap and kill surviving microbes, and are adequately digested subsequently. However, aberrant formation and disordered regulation of NETs could be implicated in the production of MPO-ANCA and subsequent development of MPA (Nakazawa et al., 2012; Ray, 2012). In addition, the extracellular DNA in NETs could accelerate MPO-ANCA production via activation of plasmacytoid dendritic cells and B cells in a toll-like receptor 9-dependent manner (Hurtado et al., 2008).

On the other hand, NETs are critically associated with thrombosis because histones within NETs can bind platelets and blood coagulants (Xu et al., 2009; Fuchs et al., 2010). NETs induce the formation of a firm thrombus with red blood cells and fibrin. Although the synergy of antimicrobial and pro-thrombotic functions of NETs is considered to be valuable in the inclusion of microbes in the NETs, excessive NETs formation conversely causes thrombosis. Thus, we focused on NETs in order to understand the association of thrombosis with MPA.

## Results and discussion

In the present case, the immediate initiation of immunosuppressive therapy was precluded because the possibility of bacterial pneumonia could not be totally ruled out. Unfortunately, the inevitable delay in the initiation of treatment could be attributed to the patient demise. Therefore, development of alternative therapeutic strategies other than immunosuppressive therapy is desirable for treatment of patients with MPA.

**Figure 2:**
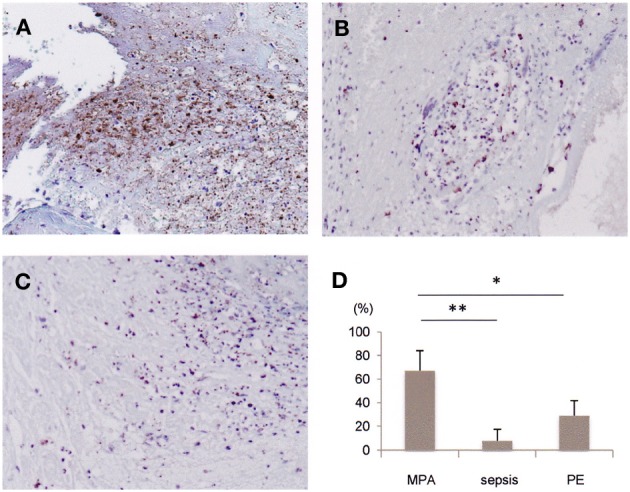
**Comparison of NETs in thrombi derived from patients with diverse diseases.** In order to detect citrullinated histones in thrombi from patients with MPA **(A)** and other diseases [**B**: bacterial sepsis, **C**: post-operative pulmonary embolism (PE)], immunohistochemistry was performed using anti-citrullinated H3 antibody. Original magnification: ×200 **(A–C)**. **(D)** Comparison on the amount of NETs among thrombi derived from patients with MPA (present case), bacterial sepsis, and post-operative PE. ^*^p < 0.05, ^**^p < 0.01.

Using the autopsy materials, we investigated the presence of NETs in the glomeruli and thrombus. As previously shown (Kessenbrock et al., 2009), NETs were identified in the glomerular crescents (Figures 1E–G). Interestingly, NETs were also identified in the thrombus (Figures 1H–J). Citrullination of histones is essential for the induction of NETs (Li et al., 2010). It is considered that histone-citrullination correlates with chromatin decondensation during NETs formation. Thus, we next investigated the degree of histone-citrullination in the thrombus. Results showed that extensive histone-citrullination was observed in the thrombus of the MPA patient (Figure 2A). When compared to other thrombi not associated with MPA, namely, the thrombi from a patient who died of bacterial sepsis (Figure 2B) and from one who died of post-operative pulmonary embolism (Figure 2C), the area of histone-citrullination was larger in the MPA patient. In order to quantify the degree, five photographs under high power view (×400) were taken at random. The area of citrullinated H3 was quantified by Image J software and then standardized by the numbers of neutrophils counted in the serial sections with hematoxylin and eosin staining. Mann-Whitney *U*-test was employed for statistical analysis. As shown in Figure 2D, the amount of NETs was significantly greater in the MPA patient in comparison with other thrombi unrelated to MPA. These findings suggest that the thrombus in the MPA patient contains abundant NETs, and thrombosis is certainly associated with MPA via NETs.

DVT can lead to fatal pulmonary embolism. However, the administration of anti-coagulants to patients with MPA should be discreet because they are prone to pulmonary hemorrhage. Based on the understanding of the role of NETs in the pathogenesis of thrombosis in MPA, active regulation of NETs could be a novel therapeutic strategy. It is known that peptidylarginine deiminase 4 (PAD4), which citrullinates histones, and nicotinamide adenine dinucleotide phosphate (NADPH) oxidase, which generates reactive oxygen species, are essential for NETs formation (Li et al., 2010; Remijsen et al., 2011). Therefore, inhibitors of these enzymes are regarded as possible candidates for active regulation of NETs. Actually, an NADPH oxidase inhibitor ameliorated the influenza A virus-induced lung inflammation in which excessive NETs were involved (Vlahos et al., 2011). Additionally, inhibitors of PAD4 and NADPH oxidase may be effective against MPA itself because NETs are involved not only in the pathogenesis of thrombosis, but also in the production of MPO-ANCA (Nakazawa et al., 2012; Ray, 2012). The inhibition of NETosis is promising for the patients with MPO-AAV.

## Concluding remarks

The quick initiation of treatment is important against MPA especially with a fulminant clinical course. The present case suggests the possibility that thrombosis and glomerulonephritis could be associated with NETosis, and that NETs could be a therapeutic target in MPA patients.

### Conflict of interest statement

The authors declare that the research was conducted in the absence of any commercial or financial relationships that could be construed as a potential conflict of interest.
